# Impact of selected single nucleotide polymorphisms in OXTR and AVPR1a genes on their expression in persons with ASD.

**DOI:** 10.1192/j.eurpsy.2023.283

**Published:** 2023-07-19

**Authors:** K. M. Wilczyński, A. Auguściak-Duma, A. Stasik, L. Cichoń, A. Sieroń, M. Janas-Kozik

**Affiliations:** 1 Department of Psychiatry and Psychotherapy of Developmental Age, Medical University of Silesia, Katowice; 2 John Paul II Paediatric Center, Sosnowiec; 3 Deparment of Molecular Biology, Medical University of Silesia, Katowice, Poland

## Abstract

**Introduction:**

Autism spectrum disorder is a heterogeneous group of disorders that affects virtually every population, regardless of their ethnic or socioeconomic origin. In recent years, the attention of researchers has been drawn to the participation of the oxytocinergic and vasopressinergic systems in the development of autism spectrum disorders. A relatively large number of studies have investigated the association of SNPs in these genes with the development of ASD, however, there is a lack of studies in the literature focusing on their actual effect on expression and on the effect of their expression on the risk of ASD.

**Objectives:**

The aim of this study was to assess the levels of expression of OXTR and AVPR1a genes and evaluate their links with both risk of ASD and genotypes of the most studied polymorphisms.

**Methods:**

The study included 132 people, 77.5% of whom were male (n = 100). 113 participants (85.6%) were diagnosed with autism spectrum disorders confirmed by the ADOS-2 test conducted by a certified diagnostician. In this group, men constituted 76.1% of the population (n = 77). The remaining 28 people did not have a diagnosis of autism spectrum disorders, and in the ADOS-2 study they obtained the result below the cut-off level. The mean age in the whole group was 14.4 years (95% CI: 13.92-14.93).

**Results:**

Significant decrease in expression of the OXTR gene was found in case of rs53576 where presence of the alternative allele (G) was linked to the 20% decrease in expression (2^(-ΔΔCt) = 0.8). In case of AVPR1a alternative allele (T) of SNP rs10877969 was linked to the 20% increase in the gene expression(2^(-ΔΔCt) = 1.197). SNPs rs2254298 (2^(-ΔΔCt) = 0.97) and rs7294536 (2^(-ΔΔCt) = 0.97) did not influence expression of the appropriate genes in significant way. In comparison between the test and control group in participants with confirmed diagnosis of ASD 13% lower expression of AVPR1a was found (2^(-ΔΔCt) = 0.87).

**Conclusions:**

Genotype of SNPs rs53576 and rs10877969 significantly influenced the levels of expression of the genes OXTR and AVPR1a respectively. In case of rs2254298 and rs7294536 observed effects were negligible. Presence of ASD diagnosis was linked to the 13% lower expression of AVPR1a. Abnormalities in AVPR1a expression seem to be more important for the development of autistic traits than the more attention-grabbing gene abnormalities for the oxytocinergic system.

**Disclosure of Interest:**

None Declared

